# Perceived quality of life in obsessive-compulsive disorder: related factors

**DOI:** 10.1186/1471-244X-6-20

**Published:** 2006-05-09

**Authors:** Beatriz Rodriguez-Salgado, Helen Dolengevich-Segal, Manuel Arrojo-Romero, Paola Castelli-Candia, Mercedes Navio-Acosta, Maria M Perez-Rodriguez, Jeronimo Saiz-Ruiz, Enrique Baca-Garcia

**Affiliations:** 1 Department of Psychiatry, Ramón y Cajal University Hospital, Carretera de Colmenar Viejo, km 9,1, 28034 Madrid, Spain; 2 Department of Psychiatry, Universidad Complutense, Ciudad Universitaria, 28040 Madrid, Spain; 3 Department of Psychiatry, Fundación Jiménez Díaz University Hospital, Avenida Reyes Catolicos 2, 28040 Madrid, Spain

## Abstract

**Background:**

Obsessive-compulsive disorder (OCD) affects young adults and has great impact on the social, emotional and work spheres.

**Methods:**

We measured perceived quality of life (QOL) in OCD patients, in order to analyse socio-demographic and clinical factors that may be associated with QOL perception. 64 OCD outpatients were assessed with the Mini International Neuropsychiatric Interview for DSM-IV, the Yale-Brown Obsessions and Compulsions scale (Y-BOCS), Hamilton's depression scale and the SF-36 self-administered global QOL perception scale.

**Results:**

We found a correlation among Hamilton's scale scores and all SF-36 subscales. The severity of the obsessive-compulsive disorder was correlated with all SF-36 subscales and with the highest scores in Hamilton's scale. The obsessions subscale was correlated to all SF-36 subscales, while the compulsions subscale was correlated only to social functioning, emotional role, mental health and vitality. Compulsions were not related to general health perception. There were significant differences between OCD patients and the Spanish general population in all SF-36 subscales except those related to physical health and pain. Gender, age, age of onset of the disorder, years of evolution and marital status of the patients did not significantly affect quality of life perception. Being employed was related to better scores in the subscale of physical role. Patients with medical comorbidity scored lower in the subscales of general health, social functioning and mental health. Patients with comorbid psychiatric disorders had worse scores in the subscales of pain, general health, social functioning and mental health.

**Conclusion:**

Quality of life perception was different in OCD patients and the general population. Quality of life perception was related to severity of the disorder, physical and psychiatric comorbidity and employment status.

## Background

The prevalence of obsessive-compulsive disorder (OCD) has been recently estimated at 2% in the general population [1-3]. Traditionally OCD was believed to be a much more uncommon disorder. It has been hypothesised that patients were reluctant to disclose OCD symptoms in medical consultations and deliberately hid them from the clinicians [4]. This meant that only the most severe cases got to be evaluated by a specialist. The high comorbidity of OCD with other psychiatric disorders (mainly anxiety and depression) has also contributed to the underdiagnosis of OCD.

OCD may not be an extremely prevalent disorder [5], but it has high clinical relevance. Its importance lies in its deep impact on the patient's life and daily activities. This is particularly relevant in OCD because this disorder mainly affects young adults, who have a potentially high level of activity in all the spheres of life. The above mentioned high comorbidity of OCD with other psychiatric disorders [6,7] also entails a significant worsening of quality of life, and has further consequences in the social and work spheres of the patient's life.

Once OCD has been diagnosed, measuring the financial costs associated with health service use would seem relatively easy. However, it is difficult to measure other factors that influence the financial impact of OCD such as impairment of quality of life and the effect of the disorder on the family, social and work environments. Since OCD is a chronic disease of relatively early onset, many of its repercussions cover the entire life span of the individual [8]. Regarding socio-economic variables, OCD patients have higher unemployment rates, lower average income, worse academic achievements and higher dependence from social security allowances and payments. According to data from the British National Survey of Psychiatric Morbidity, only 48% of OCD patients perceived a salary, 16% were unemployed and 36% were financially inactive [9]. 3% of non-institutionalised OCD patients have never had a job, suggesting an even earlier onset of the disorder. Data from North America are quite similar to these results [8]. Unfortunately there are few data from Spain.

One Spanish group [10] used the SF-36 scale and compared quality of life scores of OCD patients, general population and other groups of individuals (depressed patients, hemodialysis patients and kidney transplant receptors).

The aim of this study is to evaluate quality of life perception with the SF-36 scale in a sample of OCD outpatients, in relation to the severity of the disorder, obsessions and compulsions scales, socio-demographic data, comorbidity and age of onset of the disorder. We hypothesise that: 1) OCD patients have worse quality of life perception than the general population; 2) this is related to the severity of the clinical manifestations of the disorder; 3) the most affected areas are social functioning, emotional role and mental health.

## Methods

Between November 2002 and November 2004, we recruited 64 adult patients (older than 18) with OCD diagnosis (according to DSM-IV criteria) at the psychiatric outpatient clinic at Ramon y Cajal General Hospital. All patients were receiving psychopharmacological treatment and/or psychotherapy at the time of the study. All of them agreed to participate in the study and signed a consent form. The research carried out is in compliance with the Helsinki Declaration. The name of the body which gave approval is the Ethical Committee for Clinical Research (Comité Ético de Investigación Clínica) of Ramon y Cajal Hospital (record of meeting number 118, Research Project 042/03).

All patients were interviewed by a clinical psychiatrist and assessed with the 5.0.0 Spanish version of the Mini International Psychiatric Interview (MINI), the Hamilton scale for depression (HRSD), and the self-administered SF-36 global quality of life perception scale. The SF-36 scale was developed from the questionnaires used in the Medical Outcomes Study [11-13]. The Spanish version of the SF-36 was developed by Alonso et al [14] and validated by Ayuso-Mateos et al [15]. The psychometric values of the Spanish version of the SF-36 scale were similar to those of the original scale. The SF-36 is a self-administered questionnaire that includes 36 items in 11 multi-answer groups. It explores 8 dimensions (subscales) of health state: physical functioning, social functioning, role limitations (physical and emotional problems), mental health, vitality, pain and general health perception. One additional question explores changes in the health state during the previous year. All items and dimensions provide scores (0–100) that are directly proportional to the health state. The SF-36 detects both positive and negative health states. The normalised reference scores for the Spanish general population are shown in table 3[16].

**Table 3:** Comparison of SF-36 scores in the general Spanish population and OCD patients

SF-36 Subscales	Scores in OCD patients (n = 64)	Standard Deviation	Scores in the Spanish general population (n = 9151)	Standard Deviation	t	CI 95%	p <
Physical functioning	80.1	22.7	84.7	24.0	-1.5	(-10.5, 1.3)	0.124
Physical role	54.3	41.4	83.2	35.2	-6.5	(-37.6, -20.2)	0.001
Pain	75.6	26.2	79.0	27.9	-0.9	(-10.2, 3.5)	0.331
Global health	51.0	22.2	68.3	22.3	-6.2	(-22.8, -11.8)	0.001
Vitality	40.6	19.6	66.9	22.1	-9.5	(-31.7, -20.9)	0.001
Social functioning	48.0	32.6	90.1	20.0	-16.7	(-47.1, -37.2)	0.001
Emotional role	35.4	44.4	88.6	30.1	-14.0	(-60.6, -45.8)	0.001
Mental health	45.7	19.9	73.3	20.1	-10.9	(-32.6, -22.7)	0.001

OCD severity was evaluated with Yale-Brown's Obsessions and Compulsions Scale (Y-BOCS). Socio-demographic data were recorded for all patients, including personal and family history of psychiatric disorders, evolution of the current episode of the disorder and age of onset (see table 1).

**Table 1:** Sample description

**Variables**		**Mean (years)**	**CI (95%) (years)**
Age		36.4	33.6–37.2
Age of onset		20.7	18.5–22.9
		**%**	**N**
Gender	Male	45.3	29
	Female	54.7	35
Marital status	Single	57.8	37
	Married/living with a partner for longer than 6 months	40.6	26
	Divorced	1.6	1
Education	Primary	21.9	14
	Secondary (Technical)	17.2	11
	Secondary (Academic)	9.4	6
	University preparatory course	4.7	3
	University	46.9	30
Profession	Housewife	12.7	8
	Student	11.1	7
	Blue collar	55.6	35
	Civil servant	7.9	5
	White collar	11.1	7
Working status	Unemployed	40.6	26
	Employed	59.4	38
History of medical disease	Yes	40.6	26
	No	59.4	38
Acute Medical Disease	Yes	73.4	17
	No	26.6	47
Chronic Medical Disease	Yes	39.0	25
	No	61.0	39
Psychiatric comorbidity	Yes	46.9	30
	No	53.1	34
	Major depression	18.8	12
	Disthymia	4.7	3
	Substance abuse	14.1	9
	Anxiety disorders	9.4	6
	Other	10.9	7
	More than one comorbid psychiatric disorder	14.1	9
History of psychiatric disorder	Yes	48.4	31
	No	51.6	33
Substance abuse	Cannabis	6.2	4
	Alcohol	3.1	2
	Multiple	3.1	2
Clinical course	Insidious onset, long evolution	73.0	46
	Intermittent	17.5	11
	malignant	9.5	6

The statistical analysis was performed with the computer program SPSS 11.5. We used 2-tailed Student's t-tests (for dichotomous variables) and Analysis of Variance (ANOVA, for variables with more than 2 categories) to compare SF-36 subscales scores among the categories of categorical sociodemographic variables. We used Pearson's correlation to evaluate the correlations among the SF-36 subscales scores and the test scores (global Y-BOCS, compulsions subscale, obsessions subscale and Hamilton's Depression Scale).

## Results

### Sample description

The clinical characteristics of the sample are described in table 1.

### Measures

The mean Y-BOCS (global, compulsions and obsessions subscales) and Hamilton's scale scores are shown in table 2.

**Table 2:** Mean Y-BOCS subscales and Hamilton's scale scores in OCD patients

	**Mean score**	**SD**	**Range**
**Y-BOCS (Global)**	22.6	6.2	2–36
**Y-BOCS (Obsessions)**	11.3	3.0	0–18
**Y-BOCS (Compulsions)**	12.3	4.3	0–23
**Hamilton's Depression Scale**	9.4	5.6	1–25

The comparison of SF-36 subscales scores in OCD patients and the general population is shown in table 3. There were significant differences in all SF-36 subscales, except in those related to physical health and pain.

There were not significant differences in SF-36 subscales scores among the categories of the demographic variables gender and marital status. Age, age of onset of the disorder and years of evolution were not correlated to SF36 subscales scores. However, patients who were employed scored higher in the physical role subscale (t = -2.3; df = 62; p = 0.025). Patients with medical comorbidity had worse health perception in the subscales of general health (t = 2.1; df = 62; p = 0.037) and social functioning (t = 2.1; df = 59.4; p = 0.038). Patients with psychiatric comorbidity reported worse quality of life perception in the subscales of pain (t = 2.6; df = 62; p = 0.011), general health (t = 3.6; df = 58.4; p = 0.001), vitality (t = 2.8; df = 57.8; p = 0.007), social functioning (t = 2.6; df = 62; p = 0.012) and mental health (t = 3.2; df = 62; p = 0.002).

Y-BOCS global scores and Hamilton scale scores correlated negatively to all SF-36 subscales scores. The Y-BOCS obsessions subscale was negatively correlated to all SF-36 subscales. The Y-BOCS compulsions subscale was only negatively correlated to the social functioning, emotional role, mental health and vitality subscales of SF-36 (table 4). The SF-36 subscales that showed the weakest correlation to the Y-BOCS obsessions subscale (those related to physical functioning: physical function, physical role and pain) showed no correlation at all to the compulsions subscale. The compulsions subscale was not correlated to general health perception.

**Table 4:** Correlation among Y-BOCS, Hamilton's depression scale and SF-36 subscales scores (only statistically significant results are shown)

**SF-36**	Y-BOC (Global)*	Y-BOCS (Obsessions)*	Y-BOCS (Compulsions)*	Hamilton's depression scale*
**Physical functioning**	-0,3 p = 0,026	-0,3 p = 0,006		-0,5 p < 0,001
**Social functioning**	-0,4 p < 0,001	-0,4 p = 0,001	-0,3 p = 0,005	-0,4 p = 0,005
**Physical role**	-0,3 P = 0,014	-0,3 p = 0,024		-0,3 p = 0,014
**Emotional role**	-0,4 P = 0,004	-0,3 p = 0,031	-0,4 p = 0,002	-0,3 p = 0,029
**Mental health**	-0,4 p = 0.,001	-0,4 p = 0,003	-0,4 p = 0,002	-0,5 p < 0,001
**Vitality**	-0,3 p = 0,027	-0,4 p = 0,004	-0,3 p = 0,009	-0,5 p < 0,001
**Pain**	-0,3 p = 0,014	-0,3 p = 0,026		-0,4 p = 0,001
**General health**	-0,5 p < 0,001	-0,5 p < 0,001		-0,6 p < 0,001

* Pearson's correlation, two-tailed

We performed multiple regression analyses for all SF-36 subscales, using the SF-36 subscales scores as dependent variable and the YBOCS scores (total, compulsions and obsessions subscales) and Hamilton's scale scores as independent variables. We found that Hamilton's scale scores were negatively correlated to the SF-36 subscales related to physical functioning (physical functioning and pain), and to mental health, vitality and general health perception. YBOCS scores were only correlated to vitality and general health perception.

## Discussion

Previous studies [17] have shown that patients with anxiety and depressive disorders have higher levels of physical, social and emotional impairment than other medical or psychiatric patients.

The results of the present study are consistent with previous findings showing that OCD patients have significantly decreased mean QOL scores for every SF-36 subscale except those related to physical health and pain in comparison to the general population. However, in a recent study Moritz et al [18] observed that OCD also affected SF-36 subscales related to physical wellbeing.

We found a correlation among Hamilton scale scores and all SF-36 subscales. This is consistent with the results of other studies [18]. However, bad QOL perception in our sample may not be attributable to depressive symptoms, since the average Hamilton scale score was approximately 10 points. OCD had a clear negative repercussion on perceived QOL, independently of affective symptoms.

Sociodemographic factors, age of onset, and years of evolution of the disorder did not significantly affect QOL perception. This is consistent with the results of some studies [19]. However, other studies suggest that the delay in OCD diagnosis [20] and the length of illness [18] worsen QOL perception.

Other studies corroborate the impact of OCD on academic and work performance, social functioning and QOL [18,20-22]. We found that being employed was related to better scores in the SF-36 subscale of physical role. This stresses the importance of employment status on QOL in OCD patients.

We only found one study that assessed QOL perception in Spanish OCD patients [10], and concluded that QOL perception was worse in OCD patients than in the general population. The most affected SF-36 subscales were social functioning, emotional role and mental health. Their results agree with ours and with other recent studies [18] in that not all SF-36 subscales were equally affected.

We found that patients with medical comorbidity scored lower in the subscales of general health and social functioning. Other researchers have reported similar findings in OCD populations without medical comorbidity [10,22]. In our sample, patients with psychiatric comorbidity had worse scores in the subscales of pain, general health, vitality, social functioning and mental health. Koran et al [22] only found differences in mental health perception. The discordance in the results may be related to the low psychiatric comorbidity in Koran's sample (20%) and to the fact that all patients in our sample (but not in Koran's) [22] were receiving treatment at the time of the study. Psychiatric comorbidity may be a confounding factor since it may influence QOL perception. Previous studies have reported a negative effect of psychiatric disorders on QOL perception [23-26].

The severity of OCD was correlated with all SF-36 subscales and with the highest scores in Hamilton's scale. The obsessions subscale was correlated to all SF-36 subscales, while the compulsions subscale was correlated only to social functioning, emotional role, mental health and vitality. Compulsions were not related to general health perception. Other authors have pointed out the positive correlation between social impact and severity of the clinical manifestations of the disorder [22]. It is noteworthy that the scores in the compulsions subscale in our sample were not negatively correlated with all SF-36 subscales. Masellis et al [27] obtained similar results. This might be related to the fact that compulsions are strategies to reduce the anxiety generated by the obsessions [28]. Obsessions are perceived as intrusive and uncontrollable, generate marked uneasiness and have a greater impact on QOL than compulsions, which may be considered necessary for controlling the anxiety and discomfort. Evidence-based psychological therapies for OCD pay more attention to decreasing the compulsions than the obsessions [29], and may be less useful for patients with predominantly obsessive manifestations. Between 17% and 44% of OCD patients only experience obsessions [30]. This suggests that treatments specifically aimed at reducing the anxiety related to the obsessions may improve global QOL perception in OCD patients, particularly in those with predominant obsessive symptoms. Cognitive therapy [30-33] has been shown to be effective in patients with only obsessive symptoms, though no studies have reported an improvement in perceived QOL.

There are some limitations in our study. First, the sample size makes it difficult to control for gender and comorbidity. Second, the study design is transversal, meaning that perceived QOL may be determined by multiple punctual factors. This is magnified by the lack of control group. Third, the use of general scales (instead of specific scales for psychiatric patients) to measure perceived QOL may bias the results.

Further research is needed on perceived QOL in OCD patients, with bigger samples and control groups. Another line of investigation should be to determine the degree to which psychotherapies and pharmacological treatments make emphasis on health perception and how these treatments can improve the adaptation of OCD patients in family, social and work environments.

## Conclusion

In this sample, OCD had a clear negative repercussion on perceived QOL except in the SF-36 subscales related to physical health and pain. This suggests that not all areas of the scale were altered by the disorder.

There was a correlation among Hamilton scale scores and all SF-36 items. The severity of OCD was correlated with all SF-36 areas and with the highest scores in Hamilton's scale. The obsessions subscale was correlated to all SF-36 items, while the compulsions subscale was correlated only to social functioning, emotional role, mental health and vitality. This suggests that treatments aimed at reducing the anxiety related to obsessions may improve global QOL perception in OCD patients, particularly in those with predominant obsessive symptoms.

Sociodemographic characteristics, age of onset and years of evolution of OCD did not affect QOL perception. However, being employed was related to better scores in the area of physical role.

Patients with medical comorbidity scored lower in general health and social functioning. Patients with psychiatric comorbididity had worse scores in the areas of pain, general health, social functioning and mental health.

## Competing interests

The author(s) declare that they have no competing interests.

## Authors' contributions

BRS, HDS, MAR and PCC carried out the assessment of the sample and drafted the manuscript. MNA and JSR participated in the design and coordination of the study. MMPR participated in the statistical analysis and helped to draft the manuscript and to analyse and interpret the data. EBG conceived of the study, participated in its design and coordination and performed the statistical analysis. All authors read and approved the final manuscript.

## Pre-publication history

The pre-publication history for this paper can be accessed here:


